# Novel Applications of Intuitionistic Fuzzy Digraphs in Decision Support Systems

**DOI:** 10.1155/2014/904606

**Published:** 2014-06-16

**Authors:** Muhammad Akram, Ather Ashraf, Mansoor Sarwar

**Affiliations:** ^1^Department of Mathematics, University of the Punjab, New Campus, Lahore, Pakistan; ^2^Punjab University College of Information Technology, University of the Punjab, Old Campus, Lahore 54000, Pakistan

## Abstract

Many problems of practical interest can be modeled and solved by using graph algorithms. In general, graph theory has a wide range of applications in diverse fields. In this paper, the intuitionistic fuzzy organizational and neural network models, intuitionistic fuzzy neurons in medical diagnosis, intuitionistic fuzzy digraphs in vulnerability assessment of gas pipeline networks, and intuitionistic fuzzy digraphs in travel time are presented as examples of intuitionistic fuzzy digraphs in decision support system. We have also designed and implemented the algorithms for these decision support systems.

## 1. Introduction

Graph theory is an extremely useful tool in solving combinatorial problems in different areas including geometry, algebra, number theory, topology, operations research, optimization, computer science, engineering, and physical, biological, and social systems. Point-to-point interconnection networks for parallel and distributed systems are usually modeled by directed graphs (or digraphs). A digraph is a graph whose edges have directions and are called arcs (edges). Arrows on the arcs are used to encode the directional information: an arc from vertex (node) *x* to vertex *y* indicates that one may move from *x* to *y* but not from *y* to *x*.

Presently, science and technology are featured with complex processes and phenomena for which complete information is not always available. For such cases, mathematical models are developed to handle types of systems containing elements of uncertainty. A large number of these models are based on an extension of the ordinary set theory, namely, fuzzy sets. The notion of fuzzy sets was introduced by Zadeh [1] as a method of representing uncertainty and vagueness. Since then, the theory of fuzzy sets has become a vigorous area of research in different disciplines, including medical and life sciences, management sciences, social sciences, engineering, statistics, graph theory, artificial intelligence, signal processing, multiagent systems, pattern recognition, robotics, computer networks, expert systems, decision making, and automata theory.

Fuzzy graph theory is finding an increasing number of applications in modeling real time systems where the level of information inherent in the system varies with different levels of precision. Fuzzy models are becoming useful because of their aim of reducing the differences between the traditional numerical models used in engineering and sciences and the symbolic models used in expert systems. Kauffman's initial definition of a fuzzy graph [2] was based on Zadeh's fuzzy relations [3]. Rosenfeld [4] introduced the fuzzy analogue of several basic graph-theoretic concepts and Bhattacharya [5] gave some remarks on fuzzy graphs. Mordeson and Nair [6] defined the concept of complement of fuzzy graph and studied some operations on fuzzy graphs. In [7], the definition of complement of a fuzzy graph was modified so that the complement of the complement is the original fuzzy graph, which agrees with the crisp graph case. Atanassov [8] introduced the concept of intuitionistic fuzzy relations and intuitionistic fuzzy graphs. Akram et al. [9–11] introduced many new concepts, including strong intuitionistic fuzzy graphs, intuitionistic fuzzy hypergraphs, intuitionistic fuzzy cycles, and intuitionistic fuzzy trees. Wu [12] discussed fuzzy digraphs. In this paper, the intuitionistic fuzzy organizational, neural network models, intuitionistic fuzzy neurons in medical diagnosis, intuitionistic fuzzy digraphs in vulnerability assessment of gas pipeline networks, and intuitionistic fuzzy digraphs in travel time are presented as examples of intuitionistic fuzzy digraphs in decision support systems. Algorithms of these decision support systems are also designed and implemented.

## 2. Preliminaries

A digraph is a pair G* = (*V*, *E*), where *V* is a finite set and *E*⊆*V* × *V*. Let *G*_1_* = (*V*_1_, *E*_1_) and *G*_2_* = (*V*_2_, *E*_2_) be two digraphs. The Cartesian product of *G*_1_* and *G*_2_* gives a digraph *G*_1_* × *G*_2_* = (*V*, *E*) with *V* = *V*_1_ × *V*_2_ and

(1) E={(x,x2)⟶(x,y2) ∣ x∈V1,x2⟶y2∈E2}  ∪{(x1,z)⟶(y1,z) ∣ x1⟶y1∈E1,z∈V2}.

In this paper, we will write *xy* ∈ *E* to mean *x* → *y* ∈ *E*, and if *e* = *xy* ∈ *E*, we say *x* and *y* are adjacent such that *x* is a starting node and *y* is an ending node.

**Definition 1 (see [1, 3]).**

A fuzzy subset *μ* on a set *X* is a map *μ* : *X* → [0,1].* A fuzzy binary relation* on *X* is a fuzzy subset *μ* on *X* × *X*. By a fuzzy relation, we mean a fuzzy binary relation given by *μ* : *X* × *X* → [0,1].

**Definition 2 (see [12]).**

Let *V* be a finite set, *A* = 〈*V*, *μ*_*A*_〉 a fuzzy set of *V*, and *B* = 〈*V* × *V*, *μ*_*B*_〉 a fuzzy relation on *V*; then the ordered pair (*A*, *B*) is called a fuzzy digraph.

In 1983, Atanassov [13] introduced the concept of intuitionistic fuzzy sets as a generalization of fuzzy sets [1]. Atanassov added a new component (which determines the degree of nonmembership) in the definition of fuzzy set. The fuzzy sets give the degree of membership of an element in a given set (and the nonmembership degree equals one minus the degree of membership), while intuitionistic fuzzy sets give both a degree of membership and a degree of nonmembership which are more or less independent from each other; the only requirement is that the sum of these two degrees is not greater than 1.

**Definition 3 (see [8]).**

An intuitionistic fuzzy set (IFS) on a universe *X* is an object of the form

(2) $$\begin{array}{c} A=\left\{\langle x,\mu_{A}\left(x\right),\nu_{A}\left(x\right)\rangle x\in X\right\}, \end{array}$$

where *μ*_*A*_ (*x*)(∈[0,1]) is called degree of membership of *x* in *A*, *ν*_*A*_ (*x*)(∈[0,1]) is called degree of nonmembership of *x* in *A*, and *μ*_*A*_ and *ν*_*A*_ satisfy the following condition: for all *x* ∈ *X*,  *μ*_*A*_ (*x*) + *ν*_*A*_(*x*) ≤ 1.

**Definition 4 .**

An intuitionistic fuzzy relation *R* = (*μ*_*R*_(*x*, *y*), *ν*_*R*_(*x*, *y*)) in a universe *X* × *Y* (*R*(*X* → *Y*)) is an intuitionistic fuzzy set of the form

(3) $$\begin{array}{c} R=\left\{\langle \left(x,y\right),\mu_{A}\left(x,y\right),\nu_{A}\left(x,y\right)\rangle \;|\;\left(x,y\right)\in X\times Y\right\}, \end{array}$$

where *μ*_*A*_ : *X* × *Y* → [0,1] and *ν*_*A*_ : *X* × *Y* → [0,1]. The intuitionistic fuzzy relation *R* satisfies *μ*_*R*_ (*x*, *y*) + *ν*_*R*_ (*x*, *y*) ≤ 1 for all *x*, *y* ∈ *X*.

**Definition 5 .**

Let *R* be an intuitionistic fuzzy relation on universe *X*. Then *R* is called an intuitionistic fuzzy equivalence relation on *X* if it satisfies the following conditions:

- *R*  is intuitionistic fuzzy reflexive; that is, *R*(*x*, *x*) = (1, 0) for each *x* ∈ *X*;
- *R* is intuitionistic fuzzy symmetric; that is, *R*(*x*, *y*) = *R*(*y*, *x*) for any *x*, *y* ∈ *X*;
- *R* is intuitionistic fuzzy transitive; that is, *R*(*x*, *z*) ≥ ⋁_*y*_ (*R*(*x*, *y*)∧*R*(*y*, *z*)).

**Definition 6 .**

Let *Q* (*X* → *Y*) and *R* (*Y* → *Z*) be two intuitionistic fuzzy relations. The max-min-max composition *R*∘*Q* (*X* → *Z*) is the intuitionistic fuzzy relation defined by the membership function

(4) $$\begin{array}{c} \mu_{R\circ Q}\left(x,z\right)=\overset{}{\underset{y}{⋁}}\left(\mu_{Q}\left(x,y\right)\wedge \mu_{R}\left(y,z\right)\right) \end{array}$$

and the nonmembership function

(5) $$\begin{array}{c} \nu_{R\circ Q}\left(x,z\right)=\overset{}{\underset{y}{⋀}}\left(\nu_{Q}\left(x,y\right)\vee \nu_{R}\left(y,z\right)\right) \end{array}$$

for all (*x*, *z*) ∈ *X* × *Z* and for all *y* ∈ *Y*.

**Definition 7 .**

Let *Q* (*X* → *Y*) and *R* (*Y* → *Z*) be two intuitionistic fuzzy relations. The max-product-min-product composition *R*∘*Q* (*X* → *Z*) is the intuitionistic fuzzy relation defined by the membership function

(6) $$\begin{array}{c} \mu_{R\circ Q}\left(x,z\right)=\overset{}{\underset{y}{⋁}}\left(\mu_{Q}\left(x,y\right)\cdot \mu_{R}\left(y,z\right)\right) \end{array}$$

and the nonmembership function

(7) $$\begin{array}{c} \nu_{R\circ Q}\left(x,z\right)=\overset{}{\underset{y}{⋀}}\left(\nu_{Q}\left(x,y\right)\cdot \nu_{R}\left(y,z\right)\right) \end{array}$$

for all (*x*, *z*) ∈ *X* × *Z* and for all *y* ∈ *Y*.

Throughout this paper, we denote G*, a crisp simple digraph, and *G*, an intuitionistic fuzzy digraph.

## 3. Applications of Intuitionistic Fuzzy Digraphs in Decision Support Systems

**Definition 8 .**

An intuitionistic fuzzy digraph of a digraph G* is a pair *G* = (*A*, *B*), where *A* = 〈*V*, *μ*_*A*_, *ν*_*A*_〉 is an intuitionistic fuzzy set in *V* and *B* = 〈*V* × *V*, *μ*_*B*_, *ν*_*B*_〉 is an intuitionistic fuzzy relation on *V* such that

(8) μB(xy)≤min⁡(μA(x),μA(y)),νB(xy)≤max⁡(νA(x),νA(y)),

and 0 ≤ *μ*_*B*_ (*xy*) + *ν*_*B*_ (*xy*) ≤ 1 for all *x*, *y* ∈ *V*. We note that *B* may not be symmetric relation.

**Example 9 .**

Consider a graph G* = (*V*, *E*) such that *V* = {*v*_1_, *v*_2_, *v*_3_, *v*_4_} and *E* = {*v*_1_ *v*_2_, *v*_2_ *v*_3_, *v*_3_ *v*_4_, *v*_4_ *v*_1_}⊆*V* × *V*. Let *A* be an intuitionistic fuzzy set of *V* and let *B* be an intuitionistic fuzzy relation on *V* defined by

(9) v1v2v3v4μA0.40.60.50.5νA0.10.20.10.2v1v2v2v3v3v4v4v1μB0.10.20.30.4νB0.10.10.10.1.

**Figure 1:**
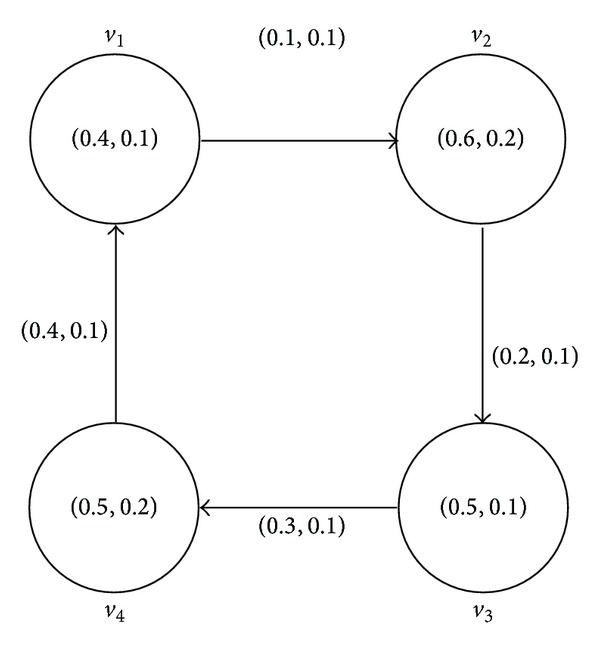
Intuitionistic fuzzy digraph.

By routine computations, it is easy to see from Figure 1 that *G* = (*A*, *B*) is an intuitionistic fuzzy digraph of G*. The intuitionistic fuzzy digraph *G* is represented by adjacency matrix given below:

(10) $$\begin{array}{c} A=\left[\begin{array}{cccc} \left(0.0,1.0\right) & \left(0.1,0.1\right) & \left(0.0,1.0\right) & \left(0.0,1.0\right)\\ \left(0.0,1.0\right) & \left(0.0,1.0\right) & \left(0.2,0.1\right) & \left(0.0,1.0\right)\\ \left(0.0,1.0\right) & \left(0.0,1.0\right) & \left(0.0,1.0\right) & \left(0.3,0.1\right)\\ \left(0.4,0.1\right) & \left(0.0,1.0\right) & \left(0.0,1.0\right) & \left(0.0,1.0\right) \end{array}\right]. \end{array}$$

We now present several applications of intuitionistic fuzzy digraphs in decision support systems in the areas of management, marketing, medical diagnosis, gas pipeline networks, and transportation.

### 3.1. Intuitionistic Fuzzy Organizational Model

In this subsection, we explore an intuitionistic fuzzy graph model to find out the most influential person within an organization, which is called influence graph. In an influence graph, vertices represent an employee and edges represent the influence of an employee on another employee of a company. Such graphs have applications in modeling social structures, communication, and distributed computing.

We consider an organization having employees and their designation as shown in Table 1. For this organization, the set of employee is *E* = {BOD, MQ, TM, MZ, AK, RB, St}.

Upon some investigation, we discover the following.

- Mujeeb Qayyum has worked with Munib Zia for over 10 years, and he values his input on strategic initiatives.
- The board of directors is chaired by a long time associate of Munib Zia. Like Mujeeb, the chair of board also values Munib.
- For reorganization, the entire marketing and HR team will be very important. Rizwan Bashir will be especially important.
- Tahir Mahmood and Rizwan Bashir have a history of conflict.
- Tahir Mahmood has great influence on the development team.

Considering the above points, an influence graph can be developed, but such a graph cannot represent the power of employees within an organization and the degree of influence of employees on each other. As the powers and influence have no defined boundaries, it is desired to represent them in the form of fuzzy set. The fuzzy digraph represents the influence of employees on each other, but there is a fair chance of the existence of nonnull hesitation part at each moment of evaluation of influence. We apply here the concept of intuitionistic fuzzy set, which is more precise about the influence and conflicts between the employees. The intuitionistic fuzzy set of the employees is as follows.

**Figure 2:**
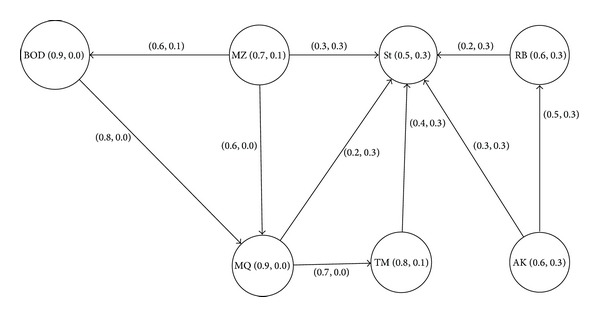
Influence intuitionistic fuzzy digraph.

We represent the influence in the intuitionistic fuzzy digraph by an edge. The resultant intuitionistic fuzzy digraph is shown in Figure 2 and corresponding adjacency matrix is shown in Table 3.

The nodes of intuitionistic fuzzy digraph in Figure 2 represent the employee and its power in terms of degree of membership and nonmembership which can be interpreted as percentage; for example, MQ possesses 90% power within the organization (Table 2). Similarly, the edges of an intuitionistic fuzzy digraph represent the influence of one person on another person, that is, end nodes of edges. The degree of membership and nonmembership can be interpreted as the percentage of positive and negative influence; for example, 60% of the time BOD works on MZ's opinion but 10% of the time they do not follow his opinion.

In Figure 2, it is clear that MZ has influence both on BOD and on MQ. He can influence both of them equally as the degree of membership in both cases is 0.6, that is, 60%. But in case of MQ, the degree of hesitation is 0.4; that is, (*π* = 1–0.6–0.0), and in case of BOQ it is 0.3; that is, (*π* = 1–0.6–0.1), which means that the hesitation in case of BOQ is more than that of MQ. But it is quite obvious that MZ is the most influential employee in the organization. Also, note that no other employee has influence on both BOD and MQ, each possessing 90% power within the organization.

### 3.2. Intuitionistic Fuzzy Neurons in Medical Diagnosis

The field of medicine is one of the most fruitful and interesting areas of application for intuitionistic fuzzy set theory. In the discrimination analysis for diagnosis of an illness, the symptoms are ranked according to the grade of discrimination of each disease by a particular symptom. A proper base knowledge is required in the medical diagnosis of a symptom. In this section, we use intuitionistic fuzzy element for knowledge base.

Consider the following set of diseases/diagnoses, *D*, and set of symptoms, *S*:

(11) D={Diabetes,Dengue,Tuberculosis},S={Temperature,Insulin,Blood  pressure,Blood  platelets,Cough}.

The intuitionistic fuzzy relation *Q* (*D* → *S*) is shown in Table 4.

Consider the set of patients as *P* = {Fayyaz, Amir, Aslam}. The intuitionistic fuzzy relation *R* (*S* → *P*) is given in Table 5.

The max product composition *T* = *R*∘*Q* is shown in Table 6.

By applying Algorithm 1 on Table 6, it is identified that Fayyaz is suffering from Dengue, Amir is suffering from Diabetes, and Aslam is a patient of Tuberculosis.

In the first portion of Algorithm 1, from lines 1–5, the parameters are set and max product composition is calculated. Each disease is ranked using *S*_*T*_; that is, *S*_*T*_ : = *μi* − *νi* ∗ *πi*, in a loop. The patient is suffering from the disease having maximum *S*_*T*_. If two diseases have the same *S*_*T*_, then their hesitation function is tested; that is, *π* = 1 − *νi*. The disease having less hesitation is selected.

### 3.3. Architecture of Intuitionistic Fuzzy Neurons in Marketability

The marketability of a book can be studied based on three criteria, that is, pictures *P*, cost *C*, and examples *E*. It is known that if a book has more examples, low cost, and a large numbers of pictures, the sale of the book improves. Suppose that by the “better SALE” we mean a sale of 60 percent of books and the pattern of the set of criteria Cr, that is, {examples, cost, pictures}, in intuitionistic fuzzy set is

(12) $$\begin{array}{c} \text{Cr}=\left[\begin{array}{ccc} \left(0.6,0.3\right) & \left(0.1,0.8\right) & \left(0.6,0.3\right) \end{array}\right]. \end{array}$$

**Figure 3:**
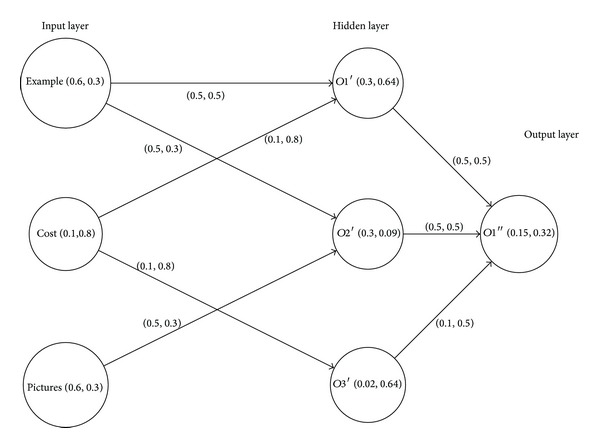
Intuitionistic fuzzy digraph of marketability.

This set can be interpreted as about 60 percent of the books contain examples and pictures, but the cost is not very low as its degree of membership is 0.1. To determine the better sale, we present it in intuitionistic fuzzy digraph given in Figure 3 and apply Algorithm 2 to it.

The digraph in Figure 3 shows a typical three-layered architecture of intuitionistic fuzzy neuron, that is, input, hidden, and output layer. In an intuitionistic fuzzy neuron, the input, hidden, and output weights are defined in terms of degree of membership and degree of nonmembership. The aggregation, or activation, of a neuron involves the degrees of both membership and nonmembership. A node in the input layer represents the criteria *C* of sales. A node in the hidden layer shows the aggregation/activation of the neuron, and the output layer shows the expected sales.

The relation between the input and hidden layers is

(13) $$\begin{array}{c} \text{IH}=\left[\begin{array}{ccc} \left(0.5,0.5\right) & \left(0.5,0.3\right) & \left(0.0,1.0\right)\\ \left(0.1,0.8\right) & \left(0.0,1.0\right) & \left(0.1,0.8\right)\\ \left(0.0,1.0\right) & \left(0.5,0.3\right) & \left(0.0,1.0\right) \end{array}\right] \end{array}$$

and the relation between the hidden and output layers is

(14) $$\begin{array}{c} \text{HO}=\left[\begin{array}{c} \left(0.3,0.64\right)\\ \left(0.3,0.09\right)\\ \left(0.02,0.64\right) \end{array}\right]. \end{array}$$

The output on the hidden layer can be computed by taking composition between IH and *C*; that is, *O*′ = *C*∘IH:

(15) $$\begin{array}{c} O^{\prime }=\left[\begin{array}{ccc} \left(0.5,0.5\right) & \left(0.5,0.3\right) & \left(0.1,0.8\right) \end{array}\right]. \end{array}$$

Similarly, the final output is calculated by taking composition between *O*′ and HO; that is, *O*′′ = *O*′∘HO:

(16) $$\begin{array}{c} O^{\prime \prime }=\left[\begin{array}{c} \left(0.15,0.32\right) \end{array}\right]. \end{array}$$

The fuzzy digraph output layer in Figure 3 shows that the sale is about 15% with 53% hesitation. Algorithm 2 describes the overall scheme.

The first three lines of Algorithm 2 set the required input. At line 4, output on hidden layer is calculated by taking the composition between *C* and IH relation. Final output is calculated on line 5 by taking the composition between output of hidden layer and HO relation. Finally, lines 7–10 check whether the results are in the desirable limits or not. If they are not within limits, the membership and nonmembership functions are modified using back propagation.

### 3.4. Intuitionistic Fuzzy Digraph in Vulnerability Assessment of Gas Pipeline Networks

Vulnerability assessment of gas network can be categorized into structural components reliability, connectivity reliability, flow performance reliability, and/or interdependent reliability. These reliabilities depended on the type of pipe and fittings used, their aging, and the connection between fitting and pipe. In most cases, we do not know the exact age and condition of connectivity. We can present these factors as an intuitionistic fuzzy set. Any gas network can be represented as an intuitionistic fuzzy digraph *G*(*F*, *P*), where *F* is the intuitionistic fuzzy set of pipe fittings, presenting their ages and connectivity conditions as degrees of membership *μ*_*F*_(*x*) and nonmembership *ν*_*F*_(*x*), and *P* is an intuitionistic fuzzy set of pipelines between fittings. In graph theoretic terms, *P* is a set of edges (i.e., pipelines) between two vertices (i.e., fittings). The degrees of membership *μ*_*P*(*xy*)_ and nonmembership *ν*_*P*(*xy*)_ are calculated as

(17) $$\begin{array}{c} \mu_{P(xy)}\leq \min\left(\mu_{F(x)},\mu_{F(y)}\right),\\ \nu_{P(xy)}\leq \max\left(\nu_{F(x)},\nu_{F(y)}\right). \end{array}$$

Consider the intuitionistic fuzzy set of pipe fittings:

(18) C1C2C3C4C5C6μF(x)0.70.50.60.70.50.5νF(y)0.10.30.30.20.40.3.

**Figure 4:**
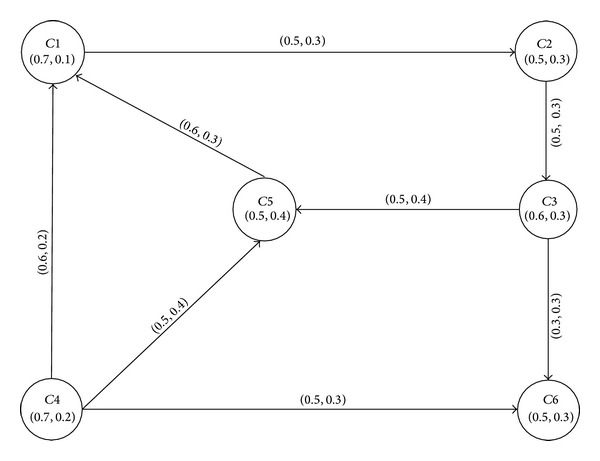
Intuitionistic fuzzy digraph of a gas pipeline network.

The intuitionistic fuzzy digraph *G*(*F*, *P*) of the gas pipeline network, shown in Figure 4, is represented by the following adjacency matrix:

(19) $$\begin{array}{c} G=\left[\begin{array}{cccccc} \left(0.0,1.0\right) & \left(0.5,0.3\right) & \left(0.0,1.0\right) & \left(0.0,1.0\right) & \left(0.0,1.0\right) & \left(0.0,1.0\right)\\ \left(0.0,1.0\right) & \left(0.0,1.0\right) & \left(0.5,0.3\right) & \left(0.0,1.0\right) & \left(0.0,1.0\right) & \left(0.0,1.0\right)\\ \left(0.0,1.0\right) & \left(0.0,1.0\right) & \left(0.0,1.0\right) & \left(0.0,1.0\right) & \left(0.5,0.4\right) & \left(0.3,0.3\right)\\ \left(0.6,0.2\right) & \left(0.0,1.0\right) & \left(0.0,1.0\right) & \left(0.0,1.0\right) & \left(0.5,0.4\right) & \left(0.5,0.3\right)\\ \left(0.6,0.3\right) & \left(0.0,1.0\right) & \left(0.0,1.0\right) & \left(0.0,1.0\right) & \left(0.0,1.0\right) & \left(0.0,1.0\right)\\ \left(0.0,1.0\right) & \left(0.0,1.0\right) & \left(0.0,1.0\right) & \left(0.0,1.0\right) & \left(0.0,1.0\right) & \left(0.0,1.0\right) \end{array}\right]. \end{array}$$

**Figure 5:**
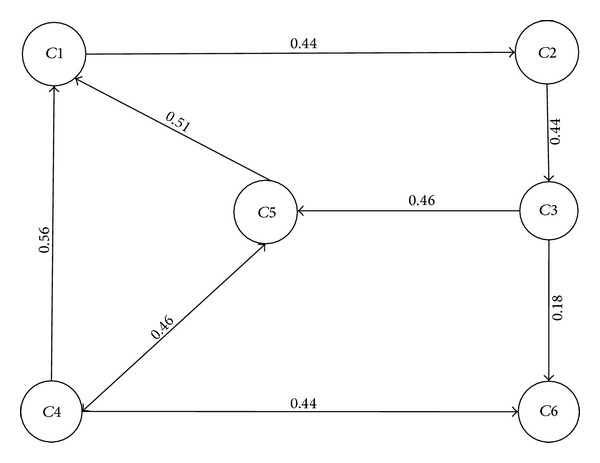
Weighted digraph of a gas pipeline network.

The final weighted digraph WG that can be used for different kind of vulnerabilities can be calculated by finding the ranks of edges as *S*_*i*_ : = *μ*_*P*_ *i*  −  *ν*_*P*_ *i* ∗ *π*_*P*_ *i*. The final adjacency matrix and weighted digraph, shown in Figure 5, are developed based on these weights:

(20) $$\begin{array}{c} \text{WG}=\left[\begin{array}{cccccc} 0 & 0.44 & 0 & 0 & 0 & 0\\ 0 & 0 & 0.44 & 0 & 0 & 0\\ 0 & 0 & 0 & 0 & 0.46 & 0.18\\ 0.56 & 0 & 0 & 0 & 0.46 & 0.44\\ 0.51 & 0 & 0 & 0 & 0 & 0\\ 0 & 0 & 0 & 0 & 0 & 0 \end{array}\right]. \end{array}$$

The overall algorithm is explained in Algorithm 3.

It takes an intuitionistic fuzzy set of pipeline fittings as an input. Lines 3–6 calculate the degrees of membership and nonmembership for edges, and line 7 assigns them to intuitionistic fuzzy set of edges and adjacency matrix is prepared in line 8. Finally, a weighted adjacency matrix is calculated in lines 9–12 using rank techniques based on the degrees of membership and nonmembership. This weighted matrix is printed in line 13 and is used for calculating vulnerability in line 14.

### 3.5. Intuitionistic Fuzzy Digraph in Travel Time

**Figure 6:**
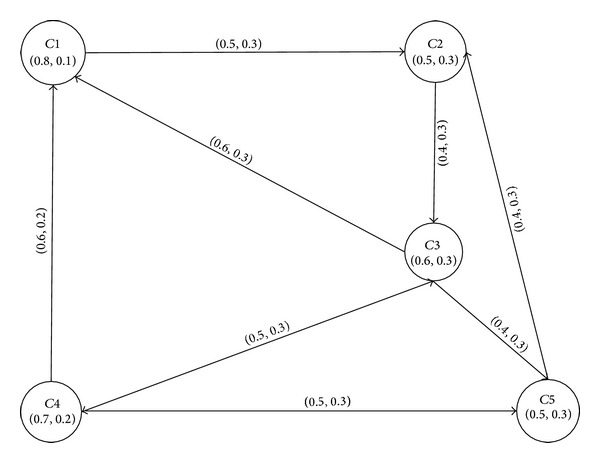
Intuitionistic fuzzy digraph of a road network.

In many network models such as transportation, communication graphs are used as a natural mathematical model to identify problems and solve them. Many of these networks can be modeled using communication graphs to find the shortest/optimal paths between the endpoints, that is, vertices and nodes, of networks. The optimality criteria are often evaluated in terms of weights of arcs/edges between two adjacent vertices in the network. In case of transportation and road networks, the travel time is mostly used as weight. The travel time is a function of the traffic density on the road and/or the length of the road. The length of a road is a crisp quantity but the traffic density is fuzzy. In a road network, we represent crossings as nodes and roads as edges. The traffic density is mostly calculated on the road between adjacent crossings. These numbers can be represented as intuitionistic fuzzy numbers.  Figure 6 shows a model of a road network represented as an intuitionistic fuzzy graph R* = (*C*, *L*), where *C* is an intuitionistic fuzzy set of crossings at which the traffic density is calculated:

(21) C={〈C1,0.8,0.1〉,〈C2,0.5,0.3〉,〈C3,0.6,0.3〉,〈C4,0.7,0.2〉,〈C5,0.5,0.3〉},

and *L* is an intuitionistic fuzzy set of roads between two crossings. The degrees of membership, *μ*_*L*(*xy*)_, and nonmembership, *ν*_*L*(*xy*)_, are calculated as

(22) $$\begin{array}{c} \mu_{L(xy)}\leq \min\left(\mu_{C(x)},\mu_{C(y)}\right),\\ \nu_{L(xy)}\leq \max\left(\nu_{C(x)},\nu_{C(y)}\right). \end{array}$$

The intuitionistic fuzzy digraph *R* of the road network is represented by the adjacency matrix given below:

(23) $$\begin{array}{c} R=\left[\begin{array}{ccccc} \left(0.0,1.0\right) & \left(0.5,0.3\right) & \left(0.0,1.0\right) & \left(0.0,1.0\right) & \left(0.0,1.0\right)\\ \left(0.0,1.0\right) & \left(0.0,1.0\right) & \left(0.4,0.3\right) & \left(0.0,1.0\right) & \left(0.0,1.0\right)\\ \left(0.6,0.3\right) & \left(0.0,1.0\right) & \left(0.0,1.0\right) & \left(0.5,0.3\right) & \left(0.4,0.3\right)\\ \left(0.6,0.2\right) & \left(0.0,1.0\right) & \left(0.5,0.3\right) & \left(0.0,1.0\right) & \left(0.5,0.3\right)\\ \left(0.0,1.0\right) & \left(0.4,0.3\right) & \left(0.0,1.0\right) & \left(0.0,1.0\right) & \left(0.0,1.0\right) \end{array}\right]. \end{array}$$

**Figure 7:**
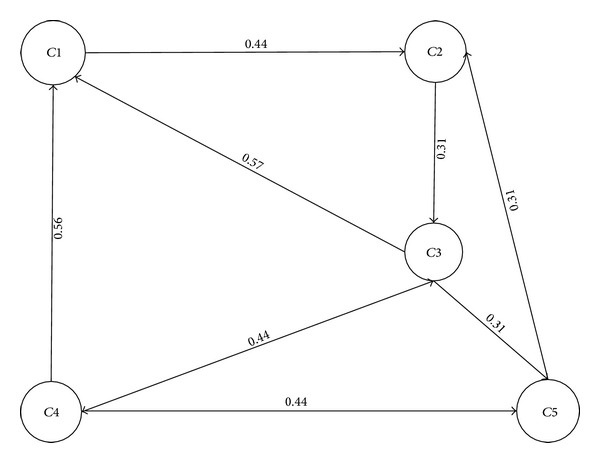
Weighted digraph of a road network.

The final weights on edges can be calculated by finding the rank as *SL*_*i*_ : = *μ*_*L*_ *i* − *ν*_*L*_ *i*∗*π*_*L*_ *i*. The final adjacency matrix and graph are developed based on these weights as shown in Figure 7:

(24) $$\begin{array}{c} \text{WR}=\left[\begin{array}{ccccc} 0 & 0.44 & 0 & 0 & 0\\ 0 & 0 & 0.31 & 0 & 0\\ 0.57 & 0 & 0 & 0.44 & 0.31\\ 0.56 & 0 & 0.44 & 0 & 0.44\\ 0 & 0.31 & 0 & 0 & 0 \end{array}\right]. \end{array}$$

The above weighted adjacency matrix represents the final weighted digraph WR, which can be used for finding the shortest/optimal path between two vertices by any of the known methods, including Djkastra and A star.  Algorithm 4 generates the weighted digraph, WR, for the given intuitionistic fuzzy graph R* and uses it to calculate the optimal path from a source node.

Algorithm 4 is quite similar to Algorithm 3. This algorithm initially sets an intuitionistic fuzzy set of crossings. Lines 3–6 calculate the values of degrees of membership and nonmembership for roads, which are assigned to intuitionistic fuzzy set of edges in line 7 and then the adjacency matrix is prepared in line 8. Finally, a weighted adjacency matrix is calculated in lines 9–12 using rank techniques based on degrees of membership and nonmembership. This weighted matrix that is printed on line 13 can be used for calculating the shortest path using any known algorithm like Djkastra or A star in line 14.

## 4. Conclusions

Fuzzy digraph theory has numerous applications in modern sciences and technology, especially in the fields of operations research, neural networks, artificial intelligence, and decision making. An intuitionistic fuzzy set is a generalization of a fuzzy set. Intuitionistic fuzzy models give more precision, flexibility, and compatibility to the system as compared to the fuzzy models. We have discussed several intuitionistic fuzzy intelligent systems in this paper. The natural extension of this research work is application of intuitionistic fuzzy digraphs in the area of soft computing including neural networks, decision making, and geographical information systems. We plan to extend our research of fuzzification to (1) application of fuzzy soft graphs in decision support systems, (2) application of rough graphs in decision support systems, and (3) application of bipolar fuzzy graphs in decision support systems.

## Figures and Tables

**Algorithm 1:**
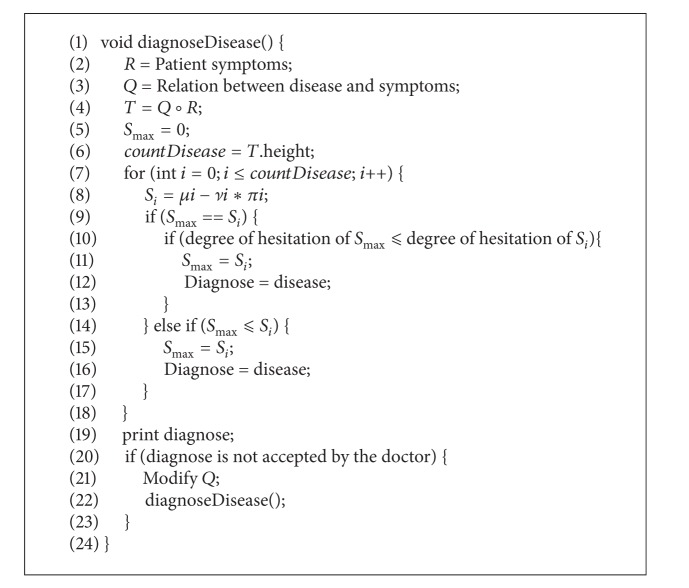
IF neurons in medical diagnosis.

**Algorithm 2:**
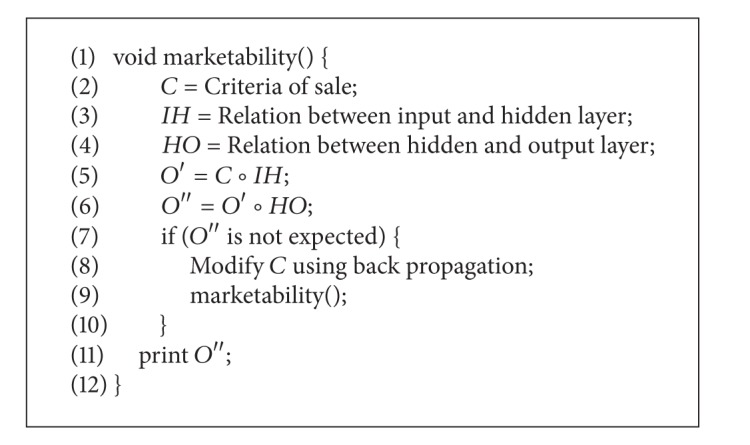
Architecture of IF neurons in marketability.

**Algorithm 3:**
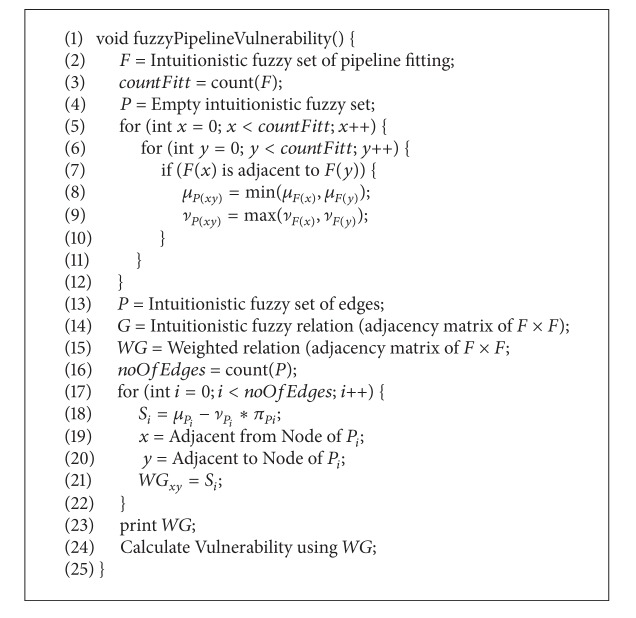
IF in vulnerability assessment of gas pipeline networks.

**Algorithm 4:**
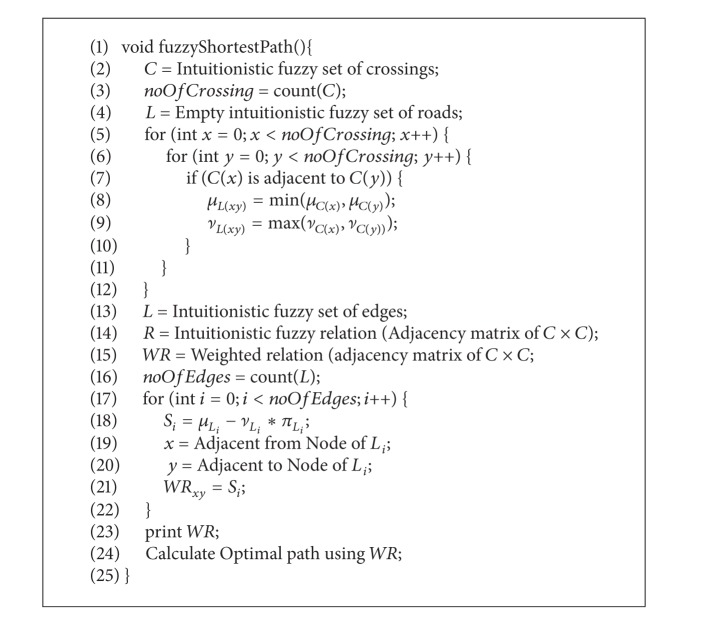
IF digraph in travel time.

**Table 1:** Names of employees in an organization and their designations.

Name	Designation
Board of Directors (BOD)	Board of Directors
Mujeeb Qayyum (MQ)	CEO
Tahir Mahmood (TM)	CTO
Munib Zia (MZ)	Director of Marketing
Arif Kaleem (AK)	Director of Product Development
Rizwan Bashir (RB)	Director of Human Resources
Staff (St)	Staff

**Table 2:** Power of employees in terms of membership degree and nonmembership degree.

	BOD	MQ	TM	MZ	AK	RB	St
*μ*_*A*_	0.9	0.9	0.8	0.7	0.6	0.6	0.5
*ν*_*A*_	0.0	0.0	0.1	0.1	0.3	0.3	0.3

**Table 3:** Adjacency matrix corresponding to Figure 2.

	BOD	MQ	TM	MZ	AK	RB	St
BOD	(0.0, 1.0)	(0.8, 0.0)	(0.0, 1.0)	(0.0, 1.0)	(0.0, 1.0)	(0.0, 1.0)	(0.0, 1.0)
MQ	(0.0, 1.0)	(0.0, 1.0)	(0.7, 0.0)	(0.0, 1.0)	(0.0, 1.0)	(0.0, 1.0)	(0.2, 0.3)
TM	(0.0, 1.0)	(0.0, 1.0)	(0.0, 1.0)	(0.0, 1.0)	(0.0, 1.0)	(0.0, 1.0)	(0.4, 0.3)
MZ	(0.6, 0.1)	(0.6, 0.0)	(0.0, 1.0)	(0.0, 1.0)	(0.0, 1.0)	(0.0, 1.0)	(0.3, 0.3)
AK	(0.0, 1.0)	(0.0, 1.0)	(0.0, 1.0)	(0.0, 1.0)	(0.0, 1.0)	(0.5, 0.3)	(0.3, 0.3)
RB	(0.0, 1.0)	(0.0, 1.0)	(0.0, 1.0)	(0.0, 1.0)	(0.0, 1.0)	(0.0, 1.0)	(0.2, 0.3)
St	(0.0, 1.0)	(0.0, 1.0)	(0.0, 1.0)	(0.0, 1.0)	(0.0, 1.0)	(0.0, 1.0)	(0.0, 1.0)

**Table 4:** Intuitionistic fuzzy relation *Q*(*D* → *S*).

*Q*	Temperature	Insulin	Blood pressure	Blood platelets	Cough
Diabetes	(0.2, 0.8)	(0.9, 0.1)	(0.1, 0.8)	(0.1, 0.8)	(0.1, 0.8)
Dengue	(0.9, 0.1)	(0.0, 0.8)	(0.8, 0.1)	(0.9, 0.1)	(0.1, 0.8)
Tuberculosis	(0.6, 0.2)	(0.0, 0.9)	(0.4, 0.4)	(0.0, 0.8)	(0.9, 0.1)

**Table 5:** Intuitionistic fuzzy relation *R*(*S* → *P*).

*R*	Fayyaz	Amir	Aslam
Temperature	(0.8, 0.1)	(0.6, 0.2)	(0.4, 0.4)
Insulin	(0.2, 0.6)	(0.9, 0.1)	(0.2, 0.7)
Blood pressure	(0.4, 0.4)	(0.1, 0.8)	(0.1, 0.7)
Blood platelets	(0.8, 0.1)	(0.2, 0.7)	(0.3, 0.6)
Cough	(0.3, 0.4)	(0.5, 0.4)	(0.8, 0.2)

**Table 6:** Composition *T* = *R*∘*Q*(*D* → *P*).

*T*	Fayyaz	Amir	Aslam
Diabetes	(0.18, 0.06)	(0.81, 0.01)	(0.18, 0.07)
Dengue	(0.72, 0.01)	(0.54, 0.02)	(0.36, 0.04)
Tuberculosis	(0.48, 0.02)	(0.45, 0.04)	(0.72, 0.02)
